# MCL-1-independent mechanisms of synergy between dual PI3K/mTOR and BCL-2 inhibition in diffuse large B cell lymphoma

**DOI:** 10.18632/oncotarget.6051

**Published:** 2015-10-09

**Authors:** J. Scott Lee, Sarah S. Tang, Veronica Ortiz, Thanh-Trang Vo, David A. Fruman

**Affiliations:** ^1^ Department of Molecular Biology and Biochemistry, University of California, Irvine, CA, USA

**Keywords:** lymphoma, apoptosis, PI3K, mTOR, BCL-2

## Abstract

The PI3K/AKT/mTOR axis promotes survival and is a frequently mutated pathway in cancer. Yet, inhibitors targeting this pathway are insufficient to induce cancer cell death as single agents in some contexts, including diffuse large B cell lymphoma (DLBCL). In these situations, combinations with inhibitors targeting BCL-2 survival proteins (ABT-199 and ABT-263) may hold potential. Indeed, studies have demonstrated marked synergy in contexts where PI3K/mTOR inhibitors suppress expression of the pro-survival protein, MCL-1. In this study, we use BH3 profiling to confirm that BCL-2 and BCL-X_L_ support survival following PI3K pathway inhibition, and that the dual PI3K/mTOR inhibitor BEZ235 strongly synergizes with BCL-2 antagonists in DLBCL. However, we identify an alternative mechanism of synergy between PI3K/mTOR and BCL-2 inhibitors, independent of MCL-1 down-regulation. Instead, we show that suppression of AKT activation by BEZ235 can induce the mitochondrial accumulation of pro-apoptotic BAD and BIM, and that expression of a constitutively active form of AKT prevents sensitization to BCL-2 antagonism. Thus, our work identifies an additional mechanism of synergy between PI3K pathway inhibitors and BCL-2 antagonists that strengthens the rationale for testing this combination in DLBCL.

## INTRODUCTION

PI3K inhibitors have recently received increased attention in blood cancers, where an inhibitor of the p110δ catalytic isoform (idelalisib) elicits significant patient responses in both chronic lymphocytic leukemia (CLL) and indolent non-Hodgkin's lymphoma [1]. However, more aggressive blood cancers such as diffuse large B cell lymphoma (DLBCL) may not respond to monotherapy with inhibitors targeting this network [2], highlighting the need to identify novel therapeutic interventions for this disease. The answer may be rational combinations. Indeed, in the activated B cell (ABC) subtype, the combination of PI3K pathway inhibitors with the BTK inhibitor, ibrutinib, has shown some promise [3, 4]. However, despite its efficacy in ABC-DLBCL, this combination fails to effectively kill the germinal center subtype (GCB) [4]. In this context, it is possible that elevated expression of BCL-2, a hallmark of the GCB subtype [5, 6], may limit the cytotoxic potential of PI3K inhibitors despite high pathway activity correlating with poor prognosis [7, 8]. Residual mTOR signaling in cells treated with selective PI3K inhibitors may also contribute to resistance [9]. These observations suggest that PI3K inhibitors or dual PI3K/mTOR inhibitors may synergize with BCL-2 antagonists in GCB-DLBCL.

The potential of simultaneously inhibiting the PI3K pathway and BCL-2 survival proteins has been previously demonstrated in several contexts [10-12]. In DLBCL sublines that are selected for resistance to the BCL-2 inhibitors ABT-199/737, dual PI3K/mTOR or mTOR selective inhibitors strongly enhance the efficacy of BCL-2 antagonism [13, 14]. In all these contexts, a decrease in MCL-1 expression, via mTORC1 inhibition [15], has been cited as the primary mechanism of synergy between PI3K and BCL-2 inhibitors. However, the PI3K pathway has several other survival outputs, particularly from AKT [16, 17], that may elicit similar drug synergy between PI3K pathway inhibitors and BCL-2 antagonists in GCB-DLBCL cells. Additionally, the effect of these drug combinations on normal lymphocytes has not yet been explored.

To gain insight into survival signaling in GCB-DLBCL, we used BH3 profiling to identify any changes in the relative balance of BCL-2 family proteins following PI3K pathway inhibition [18]. This analysis revealed that DLBCL cells become more dependent on BCL-2 and/or BCL-X_L_ for survival in the absence of PI3K pathway activity. In accord, our data show that the combination of PI3K pathway and BCL-2 inhibitors synergistically induce apoptosis in a panel of GCB-DLBCL cell lines, with dual PI3K/mTOR inhibition providing the greatest effect. Importantly, we also demonstrate synergy between PI3K/mTOR and BCL-2 inhibition in chemo-resistant DLBCL lines over-expressing BCL-2, but show that this combination lacked toxicity in normal T lymphocytes. Contrary to other tumor cell contexts, MCL-1 expression in GCB-DLBCL cells did not decrease following PI3K/mTOR inhibition. Instead, treatment with dual PI3K/mTOR inhibitors resulted in a significant accumulation of the pro-apoptotic factors BAD and BIM at the mitochondria. These effects were dependent on suppression of AKT activity, as a constitutively active mutant of AKT opposed the synergy between PI3K pathway inhibitors and BCL-2 antagonists. These findings identify a promising combination approach to achieve selective GCB-DLBCL death, and highlight a previously unpredicted mechanism of synergy.

## RESULTS

### PI3K pathway inhibition increases mitochondrial priming and enhances cytotoxicity of ABT-263 in DLBCL

To evaluate the impact of PI3K pathway inhibitors as single agents, we used several classes of chemical inhibitors targeting distinct nodes in the PI3K/AKT/mTOR axis. For each class of inhibitor, we compared the effects of two chemically distinct compounds to limit the contribution of off-target effects (Table 1). Using the minimum dose of the inhibitors required to completely inhibit their intended nodes (Supplemental Figure 1A), we confirmed that PI3K pathway suppression had little effect on viability in three GCB-DLBCL cell lines (OCI-LY1, OCI-LY8, SU-DHL4; Supplemental Figure 1B). Instead, all inhibitors caused an accumulation of cells in the G1 phase (Supplemental Figure 1C), suggestive of a cytostatic, rather than cytotoxic, response.

**Table 1:** Concentrations of PI3K pathway inhibitors used

Inhibitor	Inhibitor Class	Selective Concentration (nM)	Reference
GDC-0941	Pan-PI3K	100	[19]
ZSTK-474	Pan-PI3K	100	[20]
AKT Inhibitor VIII	Allosteric AKT	1000	[21]
MK2206	Allosteric AKT	300	[22]
MLN0128	mTOR kinase	50	[23]
AZD8055	mTOR kinase	50	[24]
Rapamycin	Allosteric mTOR	10	[25,26]
BEZ235	Dual PI3K/mTOR	50	[27]
GDC-0980	Dual PI3K/mTOR	300	[28]

**Figure 1:**
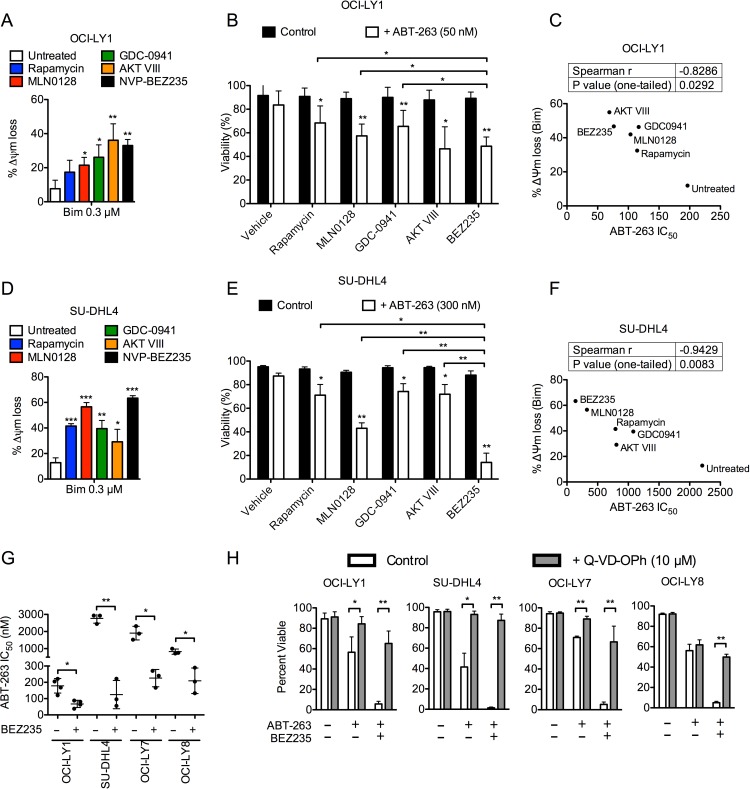
PI3K pathway inhibition increases mitochondrial priming and enhances efficacy of ABT-263 in DLBCL cell lines **A.**, **D.** OCI-LY1 and SU-DHL4 cells were treated with PI3K pathway inhibitors for 16 hours prior to permeabilization and treatment with BIM peptide (0.3μM) for 60 minutes. Mitochondrial depolarization was quantified by loss of JC-1 aggregate fluorescence; data are normalized to DMSO treated cells (*n* = 3). **B.**, **E.** OCI-LY1 and SU-DHL4 cells were treated with ABT-263 with or without PI3K pathway inhibitors for 48 hours. Viability was assessed using 7-AAD dye exclusion (*n* = 3). **C.**, **F.** Correlation between ABT-263 sensitivity (IC_50_) and MOMP induced by BIM peptide. IC_50_ is the average of three independent ABT-263 titrations in OCI-LY1 and SU-DHL4 cells treated with half-log dilutions either with or without indicated PI3K pathway inhibitor; viability was assessed by 7-AAD dye exclusion. Correlation was calculated using Spearman r and is shown above with one-tailed *P* value. **G.** ABT-263 sensitivity of four DLBCL cell lines with or without co-treatment with BEZ235. IC_50_ was obtained as described above (*n* = 3). **H.** Cells were treated with combinations of ABT-263 with BEZ235 with or without Q-VD-OPh (pan-caspase inhibitor) prior to assessing viability by 7-AAD dye exclusion (*n* = 3). All data are shown as mean ± SD. Significance was calculated using a paired one-tailed student's *t* test and is relative to untreated control unless otherwise indicated. *P < 0.05, **P < 0.005, ***P < 0.001.

Despite lacking single-agent cytotoxicity, PI3K pathway inhibitors can synergize with BCL-2 antagonists by priming cells for undergoing apoptosis [10-13]. Thus, we used BH3 profiling to measure the relative balance of apoptotic proteins following inhibition of the PI3K pathway in two GCB-DLBCL cell lines [18]. All PI3K pathway inhibitors significantly increased mitochondrial priming in these cell lines, as indicated by enhanced mitochondrial outer membrane permeabilization (MOMP) by the BIM, PUMA, and BAD peptides (Figure 1A, 1D and Supplemental Figure 2A). Interestingly, we did not observe a significant change in sensitivity to the NOXA peptide, a readout for MCL-1 modulation (Supplemental Figure 2A). Nevertheless, these data suggest that PI3K pathway inhibitors can prime DLBCL cells for undergoing apoptosis.

The heightened sensitivity to the BAD peptide (Supplemental Figure 2A) suggested that GCB-DLBCLs have an increased dependence on BAD-specific anti-apoptotic factors (e.g. BCL2 and BCL-X_L_) to maintain survival following PI3K pathway inhibition. Consistent with this interpretation, previous studies have shown that increased sensitivity to the BAD peptide correlates with higher efficacy of the dual BCL-2/BCL-X_L_ antagonist, ABT-737 [29]. Indeed, combined PI3K and BCL-2/BCL-X_L_ inhibition killed significantly more DLBCL cells compared to single-agent treatments (Figure 1B, 1E). In addition, the degree of enhanced apoptosis correlated strongly with the extent of BIM-induced MOMP (Figure 1C, 1F). Collectively, these data confirm that PI3K pathway inhibition suppresses survival signaling and sensitizes GCB-DLBCL cells to a BCL-2/BCL-X_L_ antagonist.

Among the classes of PI3K pathway inhibitors used, the dual PI3K/mTOR inhibitors, BEZ235 and GDC-0980, were consistently the most potent sensitizers to ABT-263 across several DLBCL cell lines tested (Figure 1G and Supplemental Figure 2B, 2C). Thus, we focused further experiments on the effects of dual PI3K/mTOR inhibitors. Using the median-effect method [30], we confirmed that combining BEZ235 and ABT-263 demonstrated formal synergy in both OCI-LY1 and SU-DHL4 cell lines (CI < 1, Supplemental Figure 3). To confirm the induction of apoptosis, we co-treated DLBCL cells with the pan-caspase inhibitor, Q-VD-OPh [31], which rescued the death effects of BEZ235 and ABT-263 (Figure 1H). We further confirmed that the combination induced dose- and time-dependent cleavage of caspase 3, caspase 9, and poly ADP ribose polymerase (PARP, Supplemental Figure 4), indicative of an activated apoptosis pathway. Cleavage of caspase 8 also occurred concurrently with caspase 3 cleavage, and may be the result of a positive-feedback loop [32]. Together, these data suggest that the combination of dual PI3K/mTOR and a BCL-2/BCL-X_L_ inhibitor significantly enhances the induction of apoptosis in DLBCL cell lines relative to single agent treatment.

### Combined PI3K/mTOR and BCL-2 inhibition spares normal T cells

**Figure 2:**
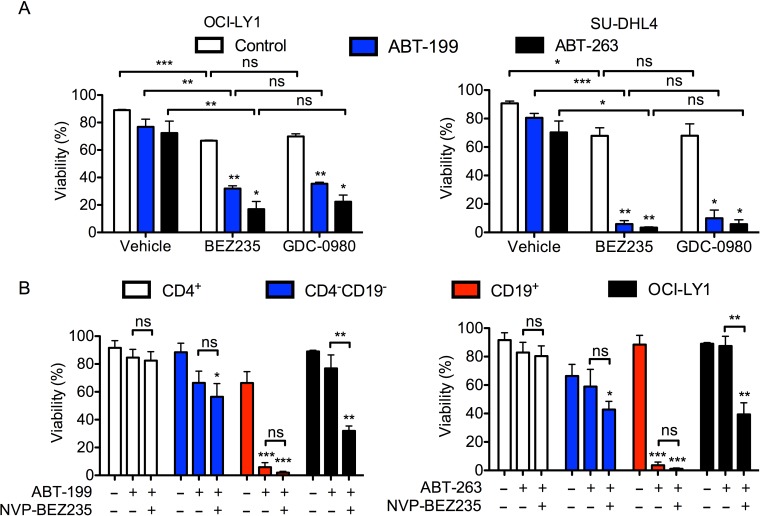
BEZ235 does not enhance the toxicity of BH3 mimetics in normal human T cells **A.** OCI-LY1 and SU-DHL4 cells were treated with ABT-263 (50 or 300 nM, respectively) or ABT-199 (5 or 50 nM, respectively) with or without BEZ235 or GDC-0980. Viability was measured using 7-AAD dye exclusion after 48 hours (*n* = 3). **B.** PBMCs were isolated from normal human blood donors and were treated with ABT-263 (30 nM) or ABT-199 (3 nM) with or without BEZ235 for 48 hours (*n* = 4). Cells were stained for CD4 and CD19 prior to assessing viability of lymphocyte subtypes by 7-AAD dye exclusion. Cells in the CD4^−^ CD19^−^ gate are mostly CD8^+^ T cells and natural killer cells. All data are shown as mean ± SD. Significance was calculated using a paired two-tailed student's t test. *P < 0.05, **P < 0.005, ***P < 0.001.

To facilitate the use of therapies combining dual PI3K/mTOR inhibitors with BCL-2 antagonists, it is necessary to consider both the efficacy and tolerability of these drugs in a preclinical setting. By inhibiting BCL-X_L_, ABT-263 results in the on-target toxicity of thrombocytopenia [33]. However, this is not observed with ABT-199, a compound that selectively inhibits BCL-2 [34]. The lack of a significant change in sensitivity to the Hrk peptide (Supplemental Figure 2A) suggested that BCL-X_L_ inhibition was dispensable for the observed synergy between ABT-263 and BEZ235. Thus, we evaluated whether the efficacy of ABT-199 could also be enhanced by the addition of PI3K/mTOR inhibitors. While ABT-199 was approximately 10-fold more potent than ABT-263 on a molar basis, the effects of both BCL-2 inhibitors at a sub-maximal concentrations were significantly enhanced by the addition of either BEZ235 or GDC-0980 in two DLBCL cell lines (Figure 2A). This suggests that suppression of BCL-2 alone is sufficient for synergy.

To further evaluate the tolerability of this combination, we determined whether it also kills normal circulating human lymphocytes, particularly CD4+ T cells, whose preservation can mediate anti-tumor immune responses and improve treatment responses [35, 36]. We isolated PBMCs from normal human donors and treated the cells with BEZ235 and either ABT-199 or ABT-263. In agreement with previous work [33], treatment with either BCL-2 antagonist alone for 48 hours was sufficient to eradicate normal B cells (CD19^+^ fraction) at low doses (Figure 2B). However, at doses of ABT-263 and ABT-199 that synergized with BEZ235 in OCI-LY1 cells, there was no significant effect on the viability of CD4^+^ T cells (Figure 2B). Importantly, the addition of BEZ235 did not further enhance the cytotoxicity of ABT-199 in CD4 T cells. Together, these data suggest that while the combination of BEZ235 and ABT-199 or ABT-263 synergistically kills both malignant and normal B cells, it does not affect normal peripheral T cells.

### Chemo-resistant DLBCL cells over-expressing BCL-2 remain sensitive to BEZ235 with ABT-199

**Figure 3:**
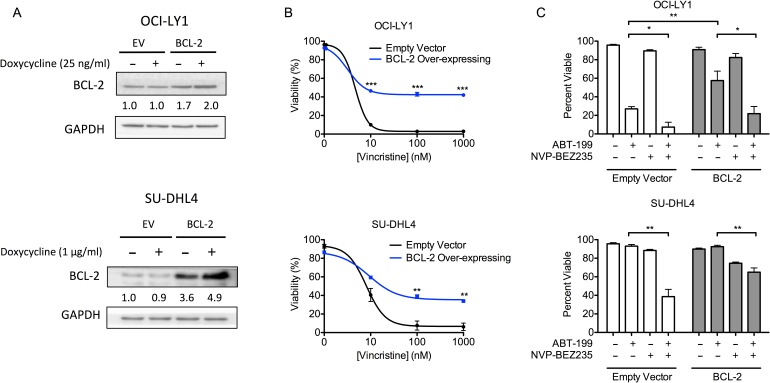
DLBCL cells over-expressing BCL-2 are resistant to a chemotherapeutic drug but remain sensitive to BEZ235 and ABT-199 **A.** Cells were treated with the indicated doxycycline doses for 24 hours. Densitometry values were normalized to GAPDH loading control, then normalized to untreated empty vector cells. Data are representative of three independent experiments. **B.** OCI-LY1 and SU-DHL4 cells expressing either empty vector or BCL-2 were treated with vincristine for 48 hours prior to assessing viability by 7-AAD dye exclusion. Cells were pre-treated with doxycycline (25 ng/ml or 1 μg/ml, respectively) for 24 hours to induce ectopic expression of BCL-2 (*n* = 3). **C.** OCI-LY1 and SU-DHL4 cells were pre-treated with doxycycline (25 ng/ml or 1 μg/ml, respectively) for 24 hours prior to treatment with ABT-199 (100 nM) with or without BEZ235 for 48 hours. Viability was assessed by 7-AAD dye exclusion (*n* = 3). All data are shown as mean ± SD. Significance was calculated using a paired one-tailed student's *t* test. *P < 0.05, **P < 0.005, ***P < 0.001.

Overexpression of BCL-2 is associated with chemo-resistance, particularly in GCB-DLBCL [5, 37]. Thus, to determine whether the combination of ABT-199 and BEZ235 could efficiently eliminate cells that over-express BCL-2, we used a two vector doxycycline-inducible system to ectopically express BCL-2 in both OCI-LY1 and SU-DHL4 cells (Figure 3A). While excessive over-expression of BCL-2 was toxic (via cleavage to a pro-apoptotic isoform, data not shown), modest increases in BCL-2 expression were sufficient to induce resistance to the chemotherapeutic agent, vincristine, in both cell lines (Figure 3B). As expected, this increased BCL-2 expression also reduced sensitivity to ABT-199 as a single agent (Figure 3C). Nevertheless, BEZ235 retained its capacity to enhance the induction of apoptosis in both cell lines (Figure 3C). Thus, combined treatment of BEZ235 and ABT-199 can efficiently kill BCL-2 over-expressing cells that are resistant to vincristine.

### Dual PI3K/mTOR inhibition does not affect MCL-1 expression in DLBCL cell lines

**Figure 4:**
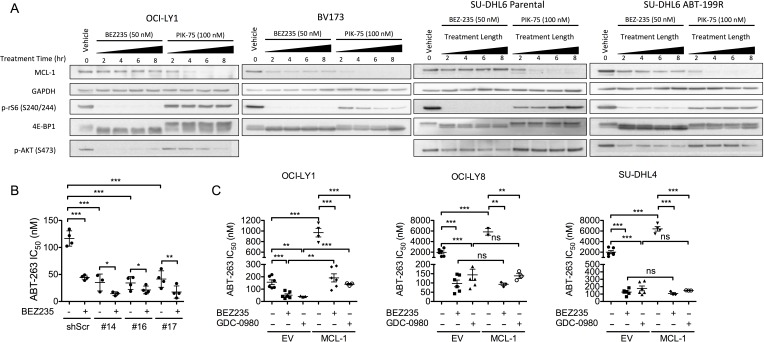
BEZ235 does not affect MCL-1 expression in OCI-LY1 cells **A.** OCI-LY1 (DLBCL), BV173 (B-cell acute lymphoblastic leukemia), and matched parental and ABT-199-resistant SU-DHL6 cells were treated with BEZ235 or PIK-75 for increasing time. Data are representative of three independent experiments. Western blots were probed with the antibodies indicated on the left. **B.** Cells stably transduced with shMCL-1 or shScramble control were treated with half-log dilutions of ABT-263 with or without BEZ235 to determine IC_50_ using GraphPad Prism software (5.0c). Data represent replicates of independent knockdown populations for each hairpin (*n* = 4). **C.** Cells stably transduced with empty vector or a doxycycline-inducible MCL-1 expression vector were pre-treated with doxycycline (1 μg/ml) for 24 hours prior to determining IC_50_ as noted above (*n* = 3). All data are shown as mean ± SD. Unless otherwise specified, significance was calculated using a paired one-tailed student's *t* test and is relative to untreated control. *P < 0.05, **P < 0.005, ***P < 0.001.

Previous studies have revealed a critical role for MCL-1 in modulating sensitivity to BCL-2/BCL-X_L_ antagonists [38-41]. Moreover, suppression of mTORC1-dependent translation has been shown to reduce the levels of MCL-1 and sensitize various cancer cells, including ABT-199 resistant DLBCL, to BCL-2 antagonists [10-13]. Thus, to examine whether mTORC1 inhibition could suppress MCL-1 expression in these DLBCL cell lines, we treated them with either BEZ235, MLN0128, or PIK-75 (a compound that suppresses MCL-1 transcription [42]) and measured MCL-1 levels over time by immunoblot. Surprisingly, treatment with BEZ235 or MLN0128 did not reduce expression of MCL-1 despite efficient suppression of both AKT and mTORC1, whereas PIK-75 fully suppressed MCL-1 expression by 8 hours (Figure 4A and Supplemental Figure 5A, 5B). In contrast, BEZ235 strongly suppressed expression of MCL-1 in a human leukemia cell line (BV173) and in ABT-199-resistant SU-DHL6 cells (Figure 4A) [13]. However, BEZ235 failed to suppress MCL-1 in matched parental SU-DHL6 cells (Figure 4A). Collectively, these data suggest that PI3K/mTOR or selective mTOR kinase inhibitors do not suppress MCL-1 expression in parental GCB-DLBCL cell lines.

We considered two possibilities to explain the observation that dual PI3K/mTOR inhibition did not reduce MCL-1 protein expression. First we tested the possibility that BEZ235 does not suppress cap-dependent translation in DLBCL cells. However, a dual-luciferase reporter assay in OCI-LY1 cells confirmed that BEZ235 significantly reduced cap-dependent translation (Supplemental Figure 5C). In addition, BEZ235 reduced the rate of total protein translation in two DLBCL cell lines as measured using a puromycin incorporation assay (SUnSET [43], Supplemental Figure 5D). Next we addressed whether MCL-1 was aberrantly stabilized, as mutations in MCL-1 may confer increased protein stability [44]. Treatment of four GCB-DLBCL lines with cycloheximide confirmed the half-life of MCL-1 to be approximately 90 minutes (Supplemental Figure 5E), in agreement with previous studies [44]. These data suggest that MCL-1 protein level is not regulated by the PI3K/mTOR network or cap-dependent translation in these DLBCL cell lines.

To further assess whether MCL-1 could contribute to the synergy between BEZ235 and BCL-2 antagonists, we tested whether modulating MCL-1 expression could alter the efficacy of the combination. Using three lentiviral shRNA constructs, we knocked down expression of MCL-1 in OCI-LY1 cells (Supplemental Figure 6A) and confirmed that reduced MCL-1 expression was sufficient to sensitize these cells to ABT-263 to a similar extent as BEZ235 (Figure 4B). However, the addition of BEZ235 further enhanced ABT-263 killing (Figure 4B), consistent with a model in which BEZ235 likely primes for apoptosis through an alternative mechanism. To determine whether uncoupling MCL-1 translation from regulation by mTORC1 could rescue from the synergy, we expressed of a form of MCL-1 lacking its endogenous 5′ UTR (Supplemental Figure 6B) [15]. Despite conferring resistance to ABT-263, ectopic expression of MCL-1 was insufficient to abolish the synergy between BEZ235 and ABT-263 in several DLBCL cell lines (Figure 4C). Thus, despite the clear influence of MCL-1 expression levels on ABT-263 sensitivity, suppression of PI3K/mTOR likely synergizes with BCL-2 antagonists through a non-MCL-1-dependent mechanism.

### PI3K pathway inhibition increases mitochondrial localization of BAD and BIM

**Figure 5:**
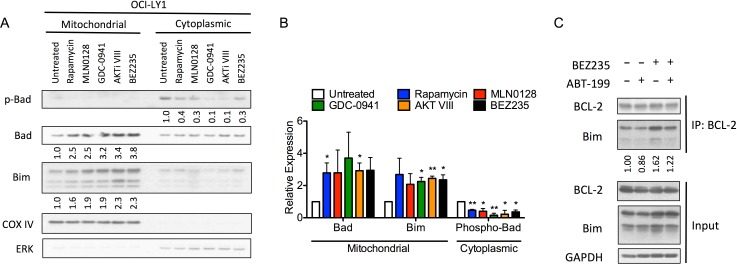
PI3K pathway inhibitors increase mitochondrial abundance of BAD and BIM **A.** Immunoblot of mitochondrial and cytoplasmic fractions of OCI-LY1 cells treated with indicated PI3K pathway inhibitors for 16 hours. Densitometry values were normalized to COX IV (mitochondrial) or ERK (cytoplasmic) loading controls, this ratio was then normalized to untreated cells. Data are representative of three independent experiments. **B.** Average densitometry values of three replicates (*n* = 3) of panel **A.**. All data are shown as mean ± SD. Significance was determined using a two-tailed one-sample t test relative to normalized control. *P < 0.05, **P < 0.005. **C.** Immunoblot of immunoprecipitation of BCL-2 (upper) or whole cell lysates (lower) following 16 hour treatment with BEZ235, ABT-199 (100 nM), or the combination. Data are representative of three independent experiments. All cells were also treated with 10 μM Q-VD-OPh to prevent cleavage of BCL-2 family proteins by caspases.

Other than suppressing mTORC1-dependent translation of pro-survival factors (e.g. MCL-1 and BCL-X_L_), inhibition of the PI3K pathway also affects the expression of several other BCL-2 family proteins [45]. Importantly, some BCL-2 family proteins are regulated by their subcellular localization [46], prompting us to examine the abundance of BCL-2 family proteins specifically at the mitochondria. This analysis revealed a significant increase in the mitochondrial abundance of pro-apoptotic BAD and BIM following PI3K pathway inhibition (Figure 5A, 5B). To confirm that these increases were functionally relevant in priming cells for apoptosis, we used immunoprecipitation to assess whether BEZ235 could increase the binding of BIM to BCL-2. BEZ235 increased both the total abundance of BIM as well as its direct binding to BCL-2 (Figure 5C), suggesting that the endogenous levels of BCL-2 are sufficient to preserve cell survival following PI3K pathway inhibition by sequestering BIM. However, the addition of ABT-199 displaced BIM from BCL-2 (Figure 5C) resulting in a net increase in un-bound BIM and induction of apoptosis [47]. These results are in agreement with the observed increase in sensitivity to the BAD peptide (Supplemental Figure 2A), which suggested that cells become more dependent on BCL-2 following treatment with BEZ235. In addition to changes in BIM expression, the reduction of cytoplasmic phospho-BAD (S136), and concomitant increase in the mitochondrial abundance of BAD, also support a model of increased dependence on BCL-2 following PI3K pathway inhibition (Figure 5A). Mitochondrial accumulation of BAD is likely a result of its binding to BCL-2 [48], suggesting that BAD may amplify the effect of BIM up-regulation by limiting the amount of free BCL-2 that can counteract BIM. Together, these data suggest that inhibition of PI3K/mTOR by BEZ235 enhances the effect of BCL-2 antagonists by increasing the abundance of BIM and BAD at the mitochondria.

### Inhibition of AKT is required for apoptotic sensitization in DLBCL cell lines

**Figure 6:**
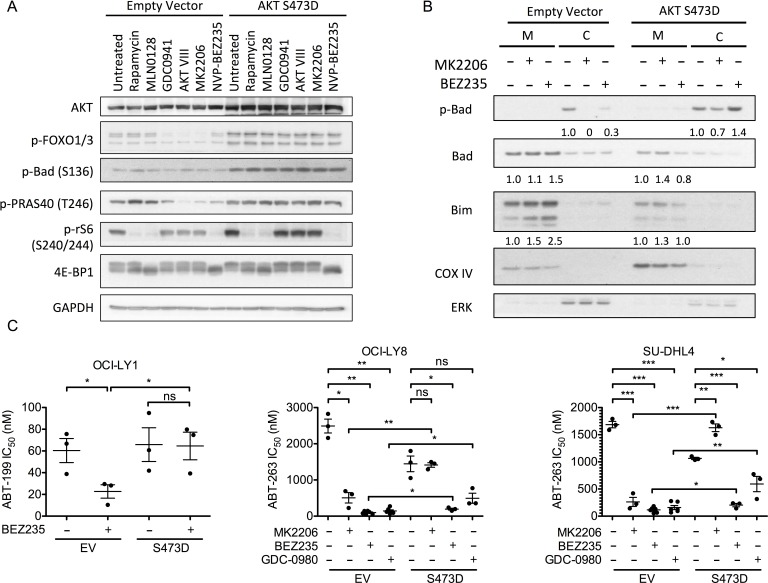
AKT suppression is a critical component of synergy between BEZ235 and ABT-199 **A.** Immunoblot of OCI-LY1 cells expressing either empty vector or phospho-mimetic AKT (S473D). Cells were pre-treated with doxycycline (1 μg/ml) for 24 hours prior to treatment with indicated PI3K pathway inhibitors for an additional 3 hours. Data are representative of three independent experiments. **B.** Immunoblot of mitochondrial (M) and cytoplasmic **C.** fractions from OCI-LY1 cells expressing empty vector or phospho-mimetic AKT (S473D). Densitometry values were normalized to COX IV (mitochondrial) or ERK (cytoplasmic) loading controls, this ratio was then normalized to untreated cells. Data are representative of three independent experiments. **C.** Sensitivity of three DLBCL cell lines expressing AKT S473D to ABT-199 in the presence or absence of MK2206, BEZ235, or GDC-0980. Cells were pre-treated with doxycycline (1 μg/ml) for 24 hours prior to treatment with increasing concentrations of ABT-199 with or without BEZ235 (50 nM) for 48 hours. Viability was assessed by 7-AAD dye exclusion and IC_50_ values were calculated using GraphPad Prism (5.0c) software (*n* = 3). All data are shown as mean ± SD. Significance was calculated using one-tailed student's *t* test *P < 0.05, **P < 0.005, ***P < 0.001.

Previous work has established that both BAD and BIM can be regulated in part by AKT [49]. Thus, to test whether sustained activation of AKT could abolish the synergy between PI3K pathway inhibitors and BCL-2 antagonists, we used a doxycycline-inducible system to express a phosphomimetic mutant of AKT (S473D). Expression of this mutant not only elevated basal AKT activity as indicated by increased phosphorylation of AKT and mTORC1 substrates, but this activity was also insensitive to PI3K or allosteric AKT inhibitors (Figure 6A and Supplemental Figure 7). Importantly, the induction of AKT S473D also completely blocked the ability of MK2206 and BEZ235 to increase the mitochondrial abundance of BIM and BAD in OCI-LY1 cells (Figure 6B). Lastly, AKT S473D expression completely abrogated the synergy between AKT inhibitors and BCL-2 inhibitors in DLBCL cells, and partially reversed the sensitization by dual PI3K/mTOR inhibitors (Figure 6C). In particular, AKT S473D expression completely protected from sensitization by BEZ235 in OCI-LY1 cells, and partially protected from GDC-0980 in OCI-LY8 and SU-DHL4 cells. Together, these data support the importance of AKT in modulating sensitivity to BCL-2 antagonists by regulating the mitochondrial abundance of BAD and BIM.

The capacity for AKT to modulate BIM expression has been attributed to direct regulation of FOXO transcription factors [16]. Thus, to confirm that AKT inhibition can activate FOXOs in DLBCL, we used a luciferase reporter assay to measure the transcriptional activity of FOXOs following PI3K pathway inhibition [16]. Treatment with either MK2206 or BEZ235 yielded significantly increased FOXO activity in control cells, but neither inhibitor significantly affected FOXO activity in OCI-LY1 cells expressing AKT S473D (Supplemental Figure 8) where phospho-FOXO levels are maintained (Figure 6A). These results support a model where activation of FOXOs downstream of AKT inhibition contributes to BEZ235-mediated apoptotic sensitization.

**Figure 7:**
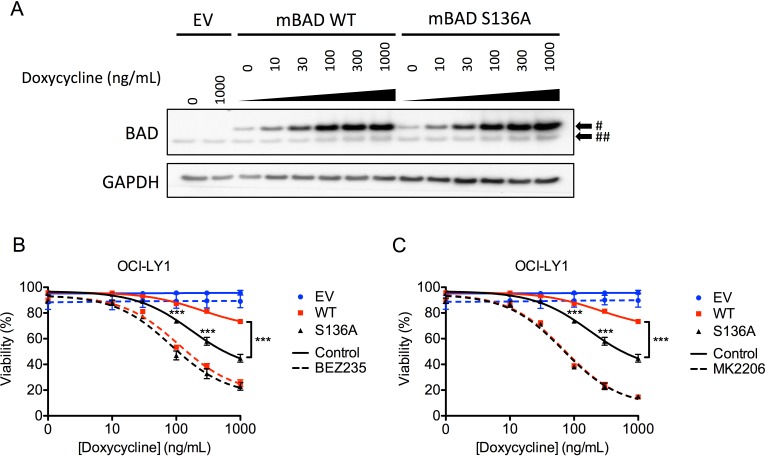
Expression of exogenous murine Bad sensitizes OCI-LY1 cells to AKT inhibition **A.** Immunoblot of mouse BAD (mBAD) induction following 24 hour treatment with indicated doses of doxycycline. Cells were also treated with 10 μM Q-VD-OPh to prevent caspase cleavage of BAD. # Indicates murine isoform, ## indicates human isoform. Data are representative of three independent experiments. **B.**, **C.** OCI-LY1 cells transduced with empty vector, mBAD wild-type (WT), and phospho-null mBAD (S136A) were treated with increasing concentrations of doxycycline ± BEZ235 **B.** or MK2206 **C.** for 48 hours. Viability was assessed using 7-AAD dye exclusion (*n* = 3). All data are shown as mean ± SD. Significance was calculated using a paired one-tailed student's *t* test ***P < 0.001.

To investigate whether suppressing AKT-mediated phosphorylation of BAD contributes to apoptotic sensitization, we inducibly expressed either wild-type or an AKT-independent, phospho-null (S136A) mutant of murine Bad (Figure 7A) [50]. While expression of either form of Bad was sufficient to induce apoptosis (Figure 7B, 7C), the phospho-null mutant induced significantly more death than wild-type Bad, despite being expressed at comparable levels (Figure 7). These data highlight the importance of AKT activity in limiting the cytotoxic potential of wild-type Bad. Despite these differences, when cells were treated with BEZ235 and MK2206, both forms of Bad induced equivalent amounts of cell death (Figures 7B, 7C). Together these data support a model where the amount of de-phosphorylated (active) Bad determines the degree of apoptosis. Overall, these data indicate inhibition of AKT, and subsequent accumulation of BAD and BIM, is a key component of the synergy between BEZ235 and ABT-199.

## DISCUSSION

**Figure 8:**
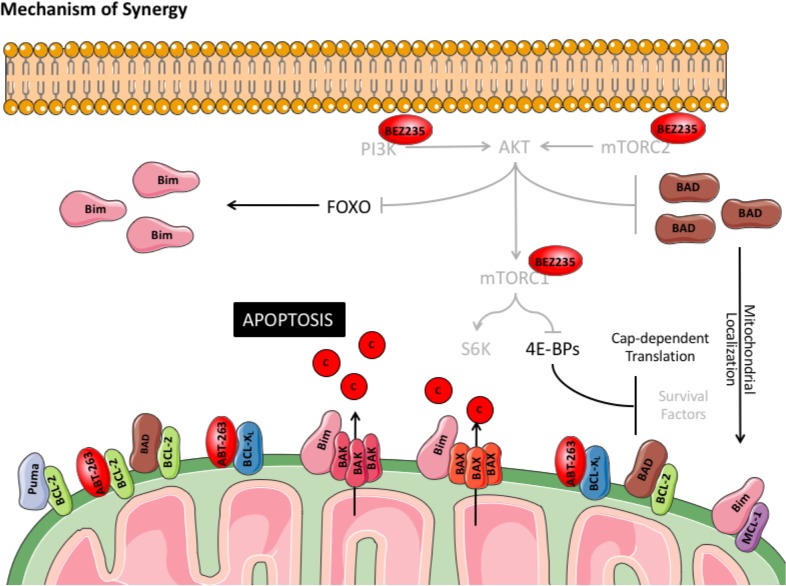
Model of synergy between BEZ235 and ABT-263 in DLBCL cell lines Treatment with BEZ235 in DLBCL cell lines completely inhibits signaling through the PI3K and downstream effectors, AKT and mTORC1. Suppression of mTORC1 may reduce cap-dependent translation of pro-survival proteins (other than MCL-1) downstream of 4E-BPs. Loss of AKT activity promotes FOXO-mediated transcription of BIM and facilitates mitochondrial accumulation of dephosphorylated BAD. When combined with ABT-263 or ABT-199, the combination promotes MOMP through BIM-activated BAX and BAK oligomerization, leading to induction of apoptosis.

Despite showing promising clinical efficacy in some blood cancers [1], PI3K/mTOR inhibitors lack single-agent cytotoxicity in aggressive diseases like DLBCL [45]. In these contexts, combination therapies may be the key to achieving cancer cell death. While promising combinations have been identified to treat the ABC subtype [3], these treatments are largely ineffective in GCB-DLBCL [4]. Contrary to ABCs where sustained BTK signaling maintains survival following PI3K pathway inhibition, in the GCB subtype, BCL-2 over-expression is the likely culprit [6]. However, the potential of simultaneous inhibition of both PI3K/mTOR and BCL-2 in this context has not been well-defined. In this study, we use BH3 profiling to confirm that BCL-2 is the critical factor that maintains GCB-DLBCL survival in the absence of PI3K pathway activity. Accordingly, combined inhibition of PI3K/mTOR and BCL-2 synergistically induced cell death, even in cells over-expressing BCL-2 or MCL-1, both of which are predictors of poor therapeutic response [5, 37, 40]. Importantly, we also show that this combination lacks toxicity in normal human T lymphocytes, which are key mediators of anti-tumor responses and are important for mediating durable responses [35, 36]. However, unlike other tumor cell contexts, where suppression of mTORC1 has been shown to reduce MCL-1 expression [10-12], our analysis of GCB-DLBCL cells reveals an unpredicted mechanism of synergy wherein suppression of AKT induces mitochondrial accumulation of BAD and BIM (Figure 8). Together, these data provide an alternative rationale for combining PI3K/mTOR and BCL-2 inhibitors as a promising therapy for GCB-DLBCL.

It is surprising that unlike in other contexts [10-12], suppression of mTORC1-dependent translation neither reduces MCL-1 expression, nor alters sensitivity to the NOXA peptide in GCB-DLBCL cell lines. A simple explanation for this discrepancy is that MCL-1 regulation may be cell-type-specific. Indeed, PI3K/mTOR inhibition significantly reduced MCL-1 expression in cells derived from a different B cell malignancy (BV173 B-ALL cells). However, work from others demonstrating the sensitivity of MCL-1 expression to PI3K/mTOR inhibitors in ABT-199/737-resistant GCB-DLBCL cells [13, 14], requires an alternative explanation. In this situation, it is plausible that the selection of ABT-199-resistant cells enriches for those cells that up-regulate MCL-1 in an mTORC1-dependent manner [40, 51, 52]. Indeed, when compared to the parental SU-DHL6 line, PI3K/mTOR inhibition selectively down-regulates MCL-1 only in ABT-199-resistant cells, highlighting the heterogeneity of survival dependencies even within variants of one cell line. It is important to note that dysregulated MCL-1 expression can be conferred by defects in any of the multiple layers of regulation (transcriptional [42, 53], translational [52], and post-translational levels [54-56]). Nevertheless, our data that knockdown or over-expression of MCL-1 can modulate sensitivity to BCL-2 antagonists strongly supports the work of others that describe the potential of targeting MCL-1 in rational combinations involving BH3 mimetics.

Contrary to previous reports, in this study we describe an alternative mechanism of synergy between PI3K/mTOR inhibitors and BCL-2 antagonists in GCB-DLBCL, which involves the mitochondrial accumulation of BAD and BIM. We identify that this effect stems from suppression of AKT, as inducible expression of constitutively active AKT prevented accumulation of BAD and BIM and abolished the synergy. While these particular survival outputs from AKT have been described previously [16, 17], the potential of their suppression in modulating sensitivity to BCL-2 antagonists has not been previously demonstrated in DLBCL. Nevertheless, it is unsurprising that accumulation of both BIM and BAD at the mitochondria can sensitize to BCL-2 antagonists. The requirement for BIM in the initiation of apoptosis has been well-characterized [57] and BIM up-regulation is required for synergy of BEZ235 and ABT-737 in ovarian cancer cells [12]. Similarly, the consequences of over-activating BAD are straightforward; since BCL-2 antagonists themselves were designed to mimic the function of BAD. By activating BAD, PI3K pathway inhibitors increase the concentration of endogenous BCL-2 antagonists (BAD and BIM protein), which can predictably enhance the effects of pharmacological BCL-2 antagonists (ABT-199/263) [11]. Despite the observed synergism between PI3K/mTOR and BCL-2 inhibitors in these GCB-DLBCL lines, it is important to note that the magnitude of BAD and BIM accumulation induced by PI3K pathway suppression is insufficient to induce apoptosis. As such, it is also unlikely that suppression of AKT would confer sensitivity to BH3 mimetics in cells that are fundamentally insensitive to BCL-2 antagonism (e.g. BAX/BAK null or MCL-1 over-expressing cells). Regardless, these data identify an alternative mechanism of synergy between PI3K/mTOR and BCL-2 inhibitors in which suppression of AKT enhances the activity/expression of pro-apoptotic factors.

In conclusion, while the mechanism may differ depending on the context, the combination of PI3K/AKT/mTOR inhibitors and BCL-2 antagonists strongly synergizes to kill DLBCL cells. In fact, the existence of multiple mechanisms of synergy may prove beneficial in combating tumor heterogeneity and preventing acquired resistance in a clinical setting. In addition, our results define alternative markers of response and identify situations in which tracking MCL-1 expression may not be predictive of patient responses. Thus, the results of this, and other, studies provide a strengthened rationale for testing dual PI3K/mTOR inhibitors with BCL-2 inhibitors in GCB-DLBCL patients.

## MATERIALS AND METHODS

### Chemicals

We obtained rapamycin, MLN0128, GDC-0941, and NVP-BEZ235 from LC Laboratories (Woburn, MA, USA); ABT-263, ABT-199, MK2206 and GDC-0980 from Active Biochem (Wan Chai, Hong Kong), and AKT inhibitor VIII from Chemdea (Ridgewood, NJ, USA). InSolution Q-VD-OPh was obtained from EMD Millipore (Billerica, MA, USA), vincristine was obtained from Sigma-Aldrich (St. Louis, MO), dimethyl sulfoxide (DMSO) from Fisher Scientific (Waltham, MA, USA) and doxycycline from Sigma-Aldrich (St. Louis, MO).

### Cell culture

OCI-LY1, OCI-LY7, OCI-LY8, and SU-DHL4 cell lines (a gift from Dr. Laura Pasqualucci, Columbia University) were cultured in IMDM (GE Healthcare Hyclone, Little Chalfont, UK) supplemented with 10% FBS, 10 mM 4-(2-hydroxyethyl)-1-piperazineethanesulfonic acid (HEPES), 10 mM L-Glutamine, 100 I.U. penicillin, and 100 μg/ml streptomycin. Cells were grown in a humidified 37°C incubator with 5% CO2. Cells were routinely tested to ensure absence of mycoplasma, and were maintained at or below 2 × 10^6^ cells/mL. Human embryonic kidney (HEK) 293T cells were cultured in Dulbecco's Modified Eagle Medium (DMEM; Life Technologies, Carlsbad, CA, USA) supplemented with 10% calf serum, 100 I.U. penicillin, and 100 μg/mL streptomycin. Human peripheral blood mononuclear cells (PBMCs) were isolated from blood samples by centrifugation through Ficoll-Paque™ (GE Healthcare, Piscataway, NJ, USA) and were grown in RPMI (Corning, NY, USA) with 10% FBS, 10 mM 4-(2-hydroxyethyl)-1-piperazineethanesulfonic acid (HEPES), 10 mM L-Glutamine, 100 I.U. penicillin, 100 μg/ml streptomycin and 55 μM BME.

### Immunoblotting

Cells were lysed in radio-immunoprecipitation assay (RIPA) buffer (150 mM NaCl, 1.0% IGEPAL^®^ CA-630, 0.5% sodium deoxycholate, 0.1% SDS, and 50 mM Tris, pH 8.0, 2 mM EDTA, 50 mM NaF) supplemented with protease inhibitor cocktail (Calbiochem, USA) and phosphatase inhibitor cocktails 2 and 3 (Sigma-Aldrich). Protein concentrations were normalized using a Bradford protein assay (Bio-Rad). Lysates were prepared at 1 μg/μl concentration in 1X XT Sample Buffer (Bio-Rad) and 5% 2-mercaptoethanol (Sigma-Aldrich). Lysates were run on 4-12% Bolt^®^ Bis-Tris Plus gels (Life Technologies), and transferred onto nitrocellulose membranes. The following antibodies were used: phospho-AKT (S473), phospho-PRAS40 (T246), phospho-rS6 (S240/244), phospho-BAD (S136), 4E-BP1, GAPDH, PARP, caspase 9, cleaved caspase 3, cleaved caspase 8, MCL-1, BIM, COX IV, ERK, phospho-FOXO1 (T24)/FOXO3 (T32), HA-Tag, BCL-X_L_, Bad (Cell Signaling Technology, Beverly, MA, USA), BCL-2 (BD Pharmingen, San Diego, CA, USA), and Bad (Santa Cruz Biotechnology, Dallas, TX, USA). The following secondary HRP-conjugated antibodies were used: anti-mouse IgG, anti-rabbit IgG (Promega, Madison, WI, USA), and Protein A (BD Pharmingen). Blots were developed using Pierce ECL Western Blotting Substrate or SuperSignal West Femto Maximum Sensitivity Substrate (Life Technologies) and detected using a Nikon D700 SLR camera as described previously [58]. Images were processed using Adobe Photoshop software and densitometry was quantified using ImageJ software.

### Cell viability

Cell viability assays were performed in 96-well plates, with 6 × 10^4^ cells in 200 μl. Cells were harvested by centrifuging the 96-well plate in a plate spinner centrifuge at 500 g for 5 minutes. Cells were incubated in 1 μg/ml 7-aminoactinomycin D (Life Technologies) in Hank's Balanced Salt Solution (HBSS; Life Technologies) supplemented with 2.5% bovine serum albumin for 10 min at room temperature. Cell fluorescence was assessed using a FACSCalibur flow cytometer (Becton-Dickinson, San Jose, CA, USA). Analysis of the data was completed using FlowJo Software v10.0.7 (TreeStar, Ashland, OR).

### BH3 profile

OCI-LY1 and SU-DHL4 cell lines were profiled as previously described, [18] with modifications. Cells were plated at 8 × 10^6^ cells per 10 ml of media and treated with inhibitors for 16 hours. 4 × 10^5^ cells were incubated in T-EB buffer (300 mM trehalose, 10 mM HEPES, 80 mM potassium chloride, 1 mM EGTA, 1 mM EDTA, 0.1% BSA, and 5 mM succinic acid) with 200 nM JC-1 (Life Technologies), 0.001% digitonin (Sigma-Aldrich), and 10 μg/ml oligomycin (Sigma-Aldrich) with either DMSO or BH3-only peptides for 60 minutes prior to analysis using a FACScalibur (Becton-Dickinson). The sequences and method of synthesis of BH3-only peptides were described previously [59]. Percent depolarization caused by each BH3-only peptide was calculated as the percent difference in the JC-1 red fluorescence (590 nm) relative to DMSO-treated control cells.

### Retro/lentiviral transductions

For all viral productions, 293T HEK cells were transfected using X-tremeGene HP DNA Transfection Reagent (Roche, Switzerland). 293T cells were incubated for 24 hours prior to replacing medium with IMDM. These virus-containing media were then harvested after an additional 24 hours and used to transduce DLBCL cell lines. For retroviral production, 293T cells were co-transfected with pCL-ampho viral packaging vector (Novus Biologicals, Littleton, CO, USA) whereas pCMV-VSVG (Addgene plasmid 8454) and psPAX2 (Addgene plasmid 12260) were co-transfected for lentivirus production. To transduce DLBCL cell lines, we incubated cells in viral supernatants for 72 hours (changing supernatant every 24 hours) with 10 μg/ml 1,5-dimethyl-1,5-diazaundecamethylene polymethobromide (polybrene, Sigma-Aldrich). Cells were treated with either blasticidin (8 μg/ml) or puromycin (2 μg/ml) for 5 days after transduction to select for stably transduced cells. Plasmid-positive cells were maintained with blasticidin (4 μg/ml) or puromycin (1 μg/ml).

### Expression plasmids

To generate DLBCL cells with doxycycline-inducible expression of a gene of interest, cells were first transduced with pMA2640 (Addgene plasmid #25434) and selected for blasticidin resistance. Expression of the improved tetracycline-controlled transactivator (rtTA-Advanced) allowed for doxycycline-inducible expression of genes downstream of the modified Tet-responsive element provided in the pLVX-tight-puro vector (Clontech). To generate MCL-1 expression plasmid, the human MCL-1 cDNA was cloned from pCMV-Flag-hMCL-1 (Addgene plasmid #25392) into pUC118 using BamHI and EcoRV. MCL-1 was then cloned into plvx-tight-puro using BamHI and NotI. To generate the AKT(S473D)-pLVX-tight-puro plasmid, we cloned AKT (S473D) from a plasmid received from Dr. Bing Su (Yale University) into pLVX-tight-puro using NotI and EcoRI. To generate BAD expression plasmids, murine Bad (S136A) in pcDNA3 (Addgene plasmid #8798) was cloned into plvx-tight-puro using EcoRI. WT murine Bad was generated using the QuikChange II XL Site-Directed Mutagenesis Kit (Agilent Technologies, Santa Clara, CA, USA) to introduce a point mutation to restore expression of a serine rather than alanine at position 136. The following primers were used for this purpose 5′-AGGACGCTCGCGTTCGGCTCCCC-3′ and 5′-GGGAGCCGAACGCGAGCGTCCT-3′. To generate BCL-2 expression plasmid, the human BCL-2 cDNA was cloned from pMIG-BCL-2 (Addgene plasmid #8793) into pLVX-tight-puro using EcoRI. pLKO.1 shRNA expression plasmids containing MCL-1 hairpins (TRCN0000005514, TRCN0000005516, TRCN0000005517) were a gift from Dr. Anand Ganesan (UC Irvine). All pLVX-tight-puro plasmids were sequenced using the following primer, 5′-AGCTCGTTTAGTGAACCGTCAGATC-3′.

### Subcellular fractionation

Subcellular fractionation was performed as described previously [60]. In brief, cells were harvested and resuspended in isotonic buffer (250 mM sucrose, 20 mM HEPES, 10 mM KCl, 1.5 mM MgCl2, 1 mM EDTA, 1 mM EGTA, 1 mM phenylmethylsulfonyl fluoride, protease inhibitor cocktail (Calbiochem, USA), and phosphatase inhibitor cocktails 2 and 3 (Sigma-Aldrich)). Cells were lysed by passing through 28 gauge insulin syringes and resulting lysates were spun at 800 g for 10 minutes at 4°C four times to remove intact cells and nuclear fractions. Supernatants were then spun at 10,000 g for 30 minutes at 4°C to separate the mitochondria-enriched heavy membrane pellet from the supernatant containing cytoplasmic fractions. Pellets were then lysed using RIPA buffer and run for immunoblotting as described above.

### Co-immunoprecipitation

Co-immunoprecipitations were performed as described previously [60]. Briefly, total cell lysates were prepared using 1% CHAPS buffer (5 mM MgCl2, 137 mM NaCl, 1 mM EDTA, 1 mM EGTA, 1% CHAPS, 20 mM Tris-HCL (pH 7.5), and protease inhibitor cocktail (Calbiochem, USA)). BCL-2 was immunoprecipitated from 500 μg protein using anti-BCL-2 (BD Pharmingen) and a slurry of protein G-Sepharose beads (GE Healthcare) at 4°C for 16 hours. Immunoprecipitates were washed three times in 1% CHAPS buffer and eluted from beads by boiling in 1X XT Sample Buffer (Bio-Rad) in 1% CHAPS buffer with 5% 2-mercaptoethanol (Sigma-Aldrich) for 10 minutes.

### Statistical analysis

The number “n” of biological replicates for each experiment is indicated in the Figure Legends. Student t-tests were applied to population means assuming equal variance (standard deviations within two-fold). The use of one- versus two-sample tests, and paired versus unpaired comparisons, was justified by the experimental design as indicated in the Figure Legends.

Additional Materials and Methods are provided in Supplementary Information.

## SUPPLEMENTARY MATERIAL FIGURES


