# Supplementary material for: MCL-1-independent mechanisms of synergy between dual PI3K/mTOR and BCL-2 inhibition in diffuse large B cell lymphoma
Source: Oncotarget. 2015 Oct 9;6(34):35202–17. doi: 10.18632/oncotarget.6051 (PMC4742099; doi:10.18632/oncotarget.6051)
Supplement: Supplementary file 1 [file oncotarget-06-35202-s001.pdf]

MCL-1-independent mechanisms of synergy between dual PI3K/mTOR and BCL-2 inhibition in diffuse large B cell lymphoma

Supplementary Material

Cell Cycle Cells were plated at 1 x 10^6 cells/ml and treated with inhibitors for 48 hours. Cells were then harvested and permeabilized in 90% ethanol. RNA was degraded using RNAse A (100 μg/ml) before staining DNA with propidium iodide (50 μg/ml) in 5 mM EDTA in 1X PBS. Fluorescence was measured using FACScalibur (Becton-Dickinson) and cell cycle populations were analyzed using FlowJo Software v10.0.7 (TreeStar).

SUnSET puromycin incorporation We performed SUnSET puromycin incorporation in OCI-LY1 and SU-DHL4 cells as previously reported [1], with modifications. Cells were plated at 3 x 10^6 cells in 3 ml and treated with inhibitors for 24 hours. Nascent peptide chains were labeled by incubating cells in 1 μg/ml puromycin (Sigma-Aldrich) for 30 minutes. Control cells were co-treated with 20 μg/ml cycloheximide (Sigma-Aldrich) during puromycin labeling. Lysates from these cells were run for immunoblotting as described above. An anti-puromycin primary antibody (EMD Millipore, Billerica, MA, USA) was used and visualized using chemiluminescence after incubation with an anti-mouse HRP-conjugated secondary (Promega).

Luciferase Assays For measurement of FOXO transcriptional activity, cells were co-transfected with equal amounts of the pRL-TK plasmid (Promega) and the FOXO3 Firefly luciferase reporter plasmid (gift from Dr. Anne Brunet, Stanford University). For cap-dependent translation studies, the pRSTF-CVB3 dual-luciferase reporter plasmid was used as described previously [2]. For all experiments, cells were transfected using a GenePulser Xcell™ (Bio-Rad) at 280 V and 0.975 F. All cells were treated with indicated inhibitors for 16 hours, prior to harvesting. Luciferase activity was measured using substrates from the Dual-Luciferase Reporter

Assay System kit (Promega) and a Sirius single tube luminometer (Titertek-Berthold, Pforzheim, Germany).

Power Analysis Power analysis was performed using tools at www.danielsoper.com/statcalc3. A minimum sample size of 3 was chosen based on power analysis, to detect an effect size of 3.0 with alpha level of 0.8 and probability of 0.05. The effect size of 3.0 was determined based on assumed viability data in which the control group 1 has a mean of 100 and SD of 10, and test group 2 has a mean of 70 and SD of 10.

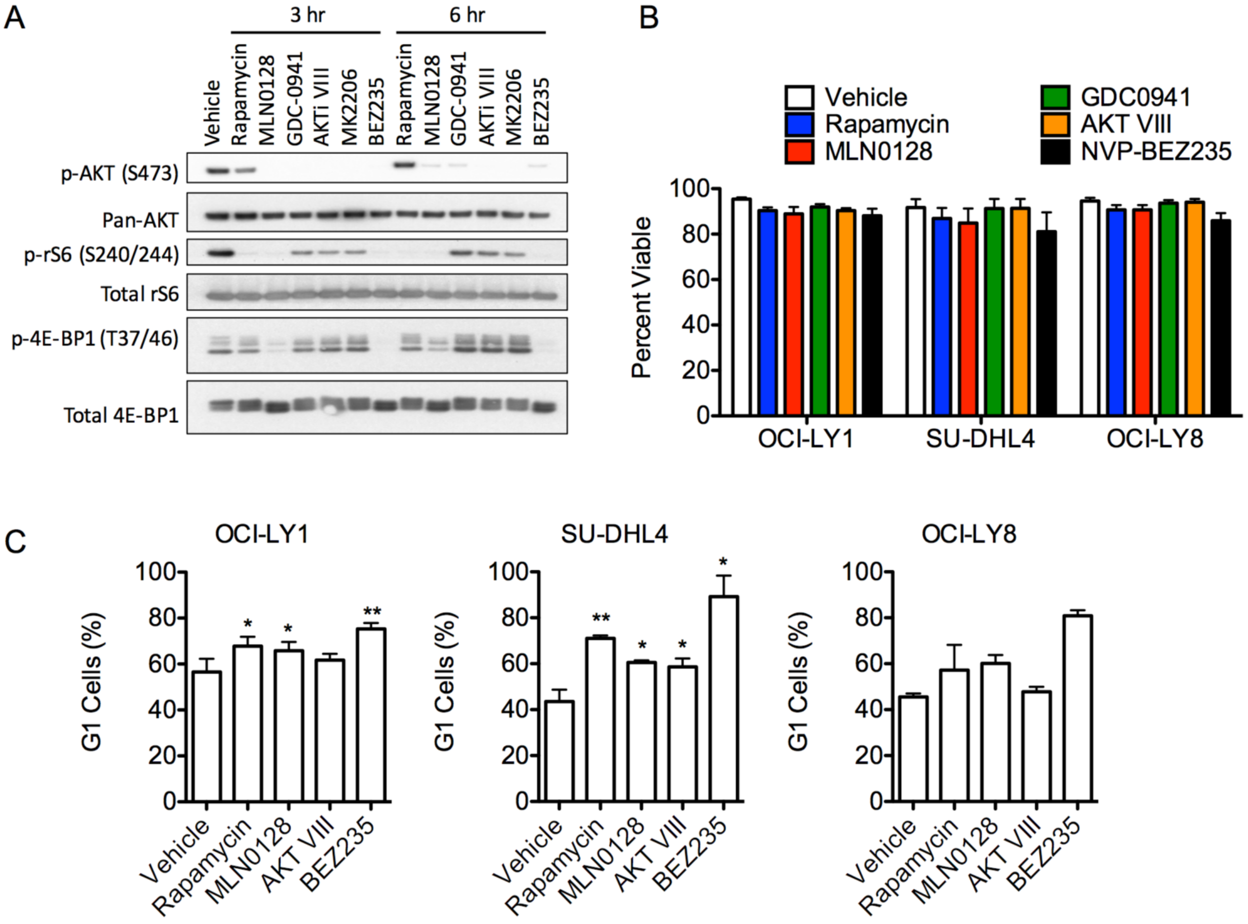

Supplemental Figure 1. Suppression of the PI3K/AKT/mTOR pathway components is cytostatic in DLBCL. (A) Immunoblot analysis of OCI-LY1 cells with indicated antibodies. Cells were treated with indicated inhibitors (concentrations indicated in Table 1) for 3 or 6 hours. Data are representative of three independent experiments. (B) Three DLBCL cell lines (OCI-LY1, SU-DHL4, and OCI-LY8) were treated with all inhibitors at doses indicated above for 48 hours prior to measuring viability by 7-AAD dye exclusion using flow cytometry (n = 3). (C) Cells were treated with all inhibitors at doses indicated above for 48 hours before determining cell cycle profile using propidium iodide (PI) staining. Percentage of cells in the G1 stage of cell cycle was obtained using FlowJo (v10) software (n = 3 for OCI-LY1 and SU-DHL4, n = 2 for OCI-LY8). All data are shown as mean ± SD. Significance was calculated using a paired one-tailed student's t test and is relative to untreated control. *P < 0.05, **P < 0.005, ***P < 0.001.

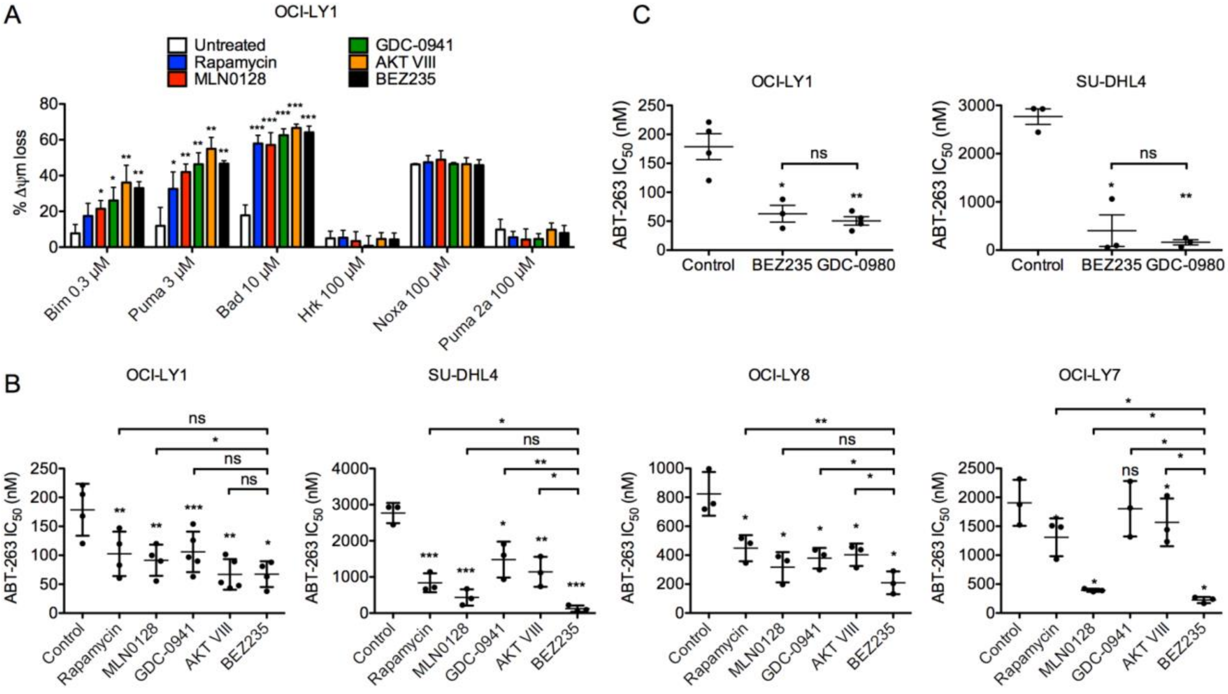

Supplemental Figure 2. Dual PI3K/mTOR inhibition consistently demonstrates greatest enhancement of ABT-263 efficacy. (A) OCI-LY1 cells were treated with various PI3K pathway inhibitors for 16 hours prior to permeabilization and treatment for 60 minutes with the BH3-only peptides indicated on the x-axis (Hrk and NOXA are BCL-X_L and MCL-1 specific antagonists, respectively). Mitochondrial depolarization was quantified by loss of JC-1 aggregate fluorescence; data are normalized to DMSO treated cells. Note that Puma 2a is mutated to no longer bind to BCL-2 family proteins and serves as a negative control (n = 3). (B) Sensitivity of four DLBCL cell lines to ABT-263 in the presence or absence of various PI3K pathway inhibitors. Viability was assessed using 7-AAD dye exclusion after 48 hours and IC50 values were calculated using GraphPad Prism software (n = 4 for OCI-LY1, n = 3 for SU-DHL4, OCI-LY8, and OCI-LY7). (C) Sensitivity of two DLBCL cell lines to ABT-263 in combination with two chemically distinct dual PI3K/mTOR inhibitors (BEZ235 and GDC-0980). Viability and IC50 was assessed as described above (n = 4 for OCI-LY1, n = 3 for SU-DHL4). All data are shown as mean ± SD. Significance was calculated using one-tailed student's t test and is relative to untreated control unless otherwise indicated. *$P < 0.05$, **$P < 0.005$, ***$P < 0.001$.

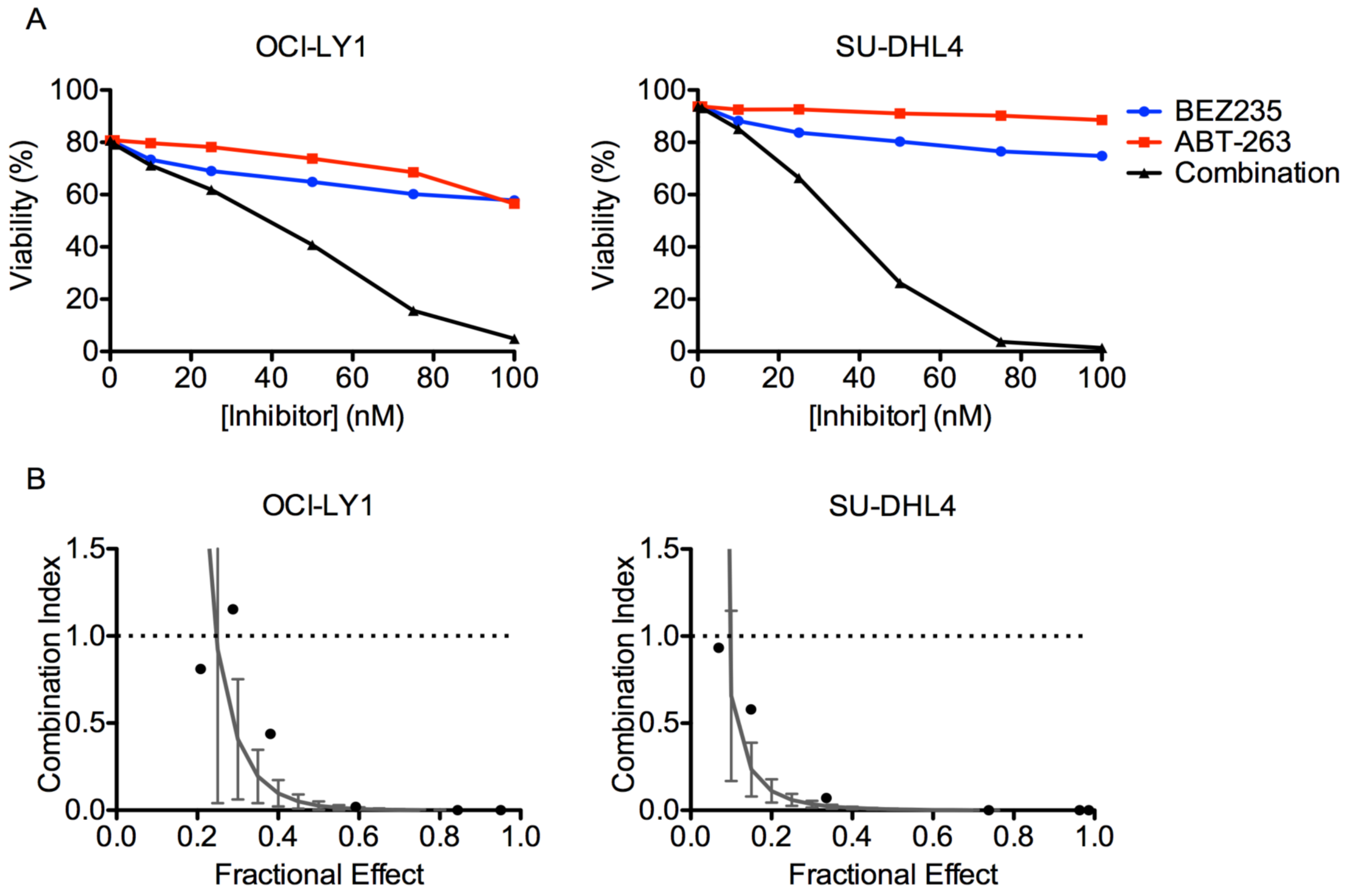

Supplemental Figure 3. BEZ235 and ABT-263 synergistically kill DLBCL cell lines. (A) OCI-LY1 and SU-DHL4 cells were treated with indicated doses of inhibitors for 48 hours prior to assessing viability by 7-AAD dye exclusion (note that concentration for ABT-263 in the SU-DHL4 cells is 6-fold higher than the indicated dose). Combination treatments in the OCI-LY1 cells were at a constant 1:1 ratio whereas SU-DHL4 cells were treated at a 1:6 ratio. Similar effects were observed with different ratios and concentration ranges in both cell lines. (B) Predicted combination indexes for OCI-LY1 and SU-DHL4 treated with BEZ235 and ABT-263 using CalcuSyn software (CI > 1 antagonism, CI = 1 additivity, CI < 1 synergy). Data were normalized to untreated control and converted to fractional effect as the inverse of viability (fractional effect of 1 is synonymous with 0% viability). Black circles are experimental data from panel (A).

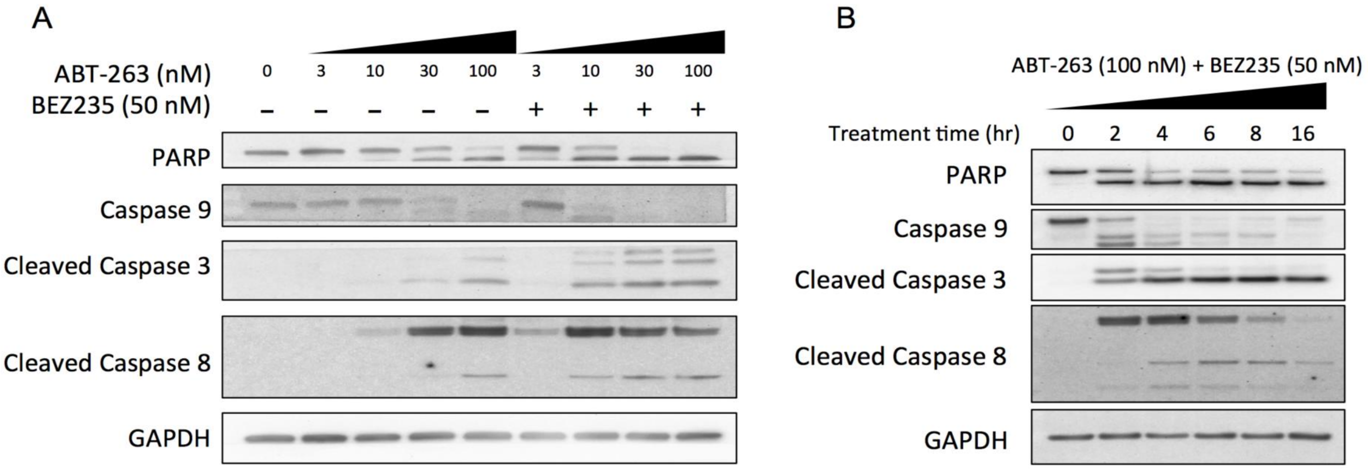

Supplemental Figure 4. Combining BEZ235 and ABT-263 induces caspase and PARP cleavage. (A) OCI-LY1 cells were treated with increasing doses of ABT-263 with or without BEZ235 for 6 hours prior to immunoblot analysis of caspase and PARP cleavage. (B) OCI-LY1 cells were treated with ABT-263 (100 nM) in combination with BEZ235 (50 nM) for indicated time prior to immunoblot analysis of caspase and PARP cleavage. Data are representative of three independent experiments.

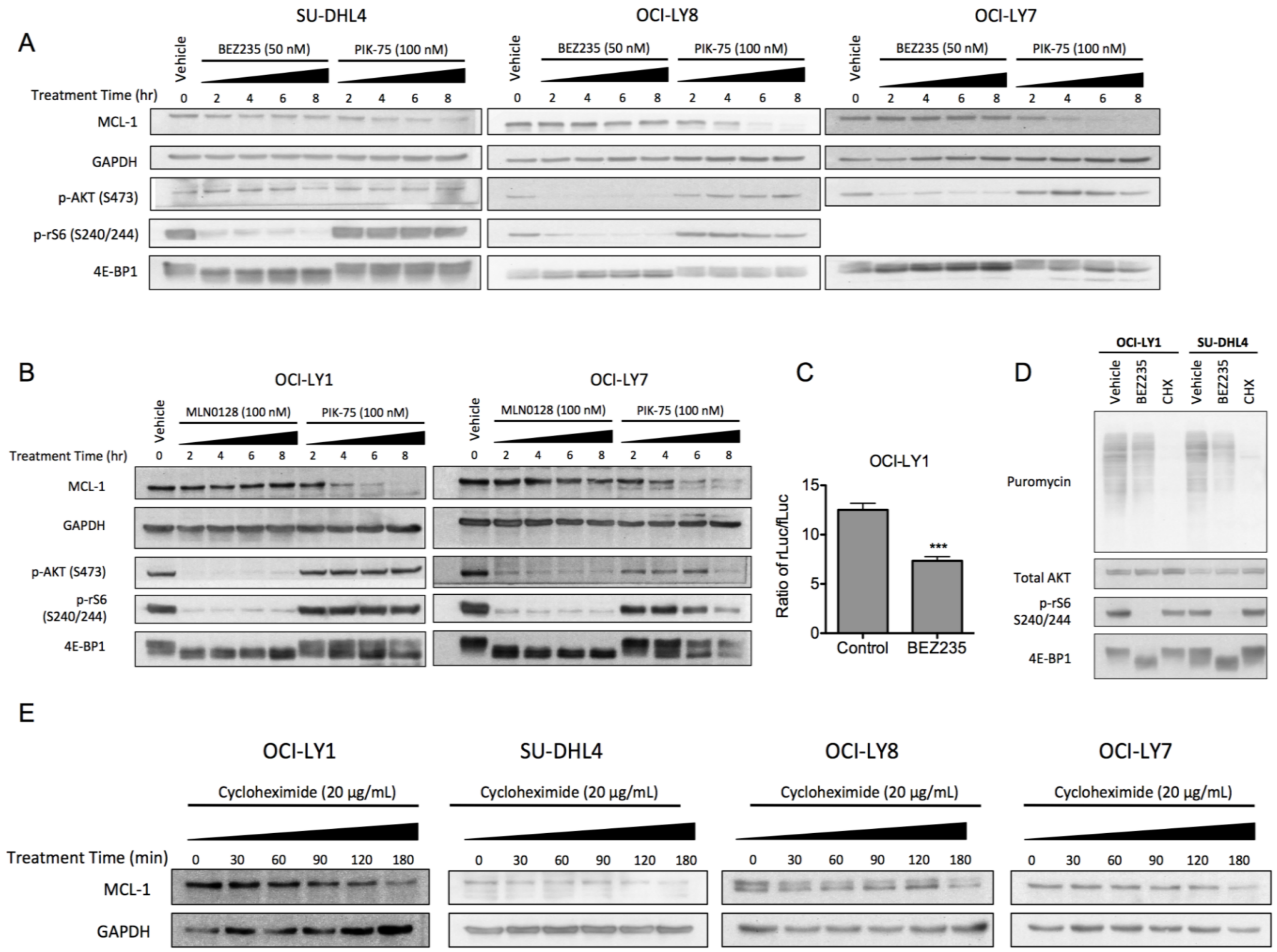

Supplemental Figure 5. BEZ235 does not affect MCL-1 expression in DLBCL cell lines. (A,B) SU-DHL4, OCI-LY8, and OCI-LY7 cell lines were treated with PIK-75 and either BEZ235 (A) or MLN0128 (B), at the concentrations indicated. Data are representative of three independent experiments. (C) Cells were transfected with reporter construct then treated with BEZ235 for 16 hours. The ratio of Renilla (cap-dependent) to Firefly (IRES-dependent) luciferase activity was determined by luminometer (n = 3). (D) Cells were pre-treated with vehicle (DMSO) or BEZ235 for 6 hours prior to pulse treatment of puromycin (1 µg/ml) for 30 minutes, or co-treatment with cycloheximide (CHX, 20 µg/ml) and puromycin for 30 minutes. Representative of two independent experiments. (E) Four DLBCL cell lines were treated with cycloheximide (20 µg/ml) for indicated treatment time prior to harvesting and lysis. Data are representative of three independent experiments.

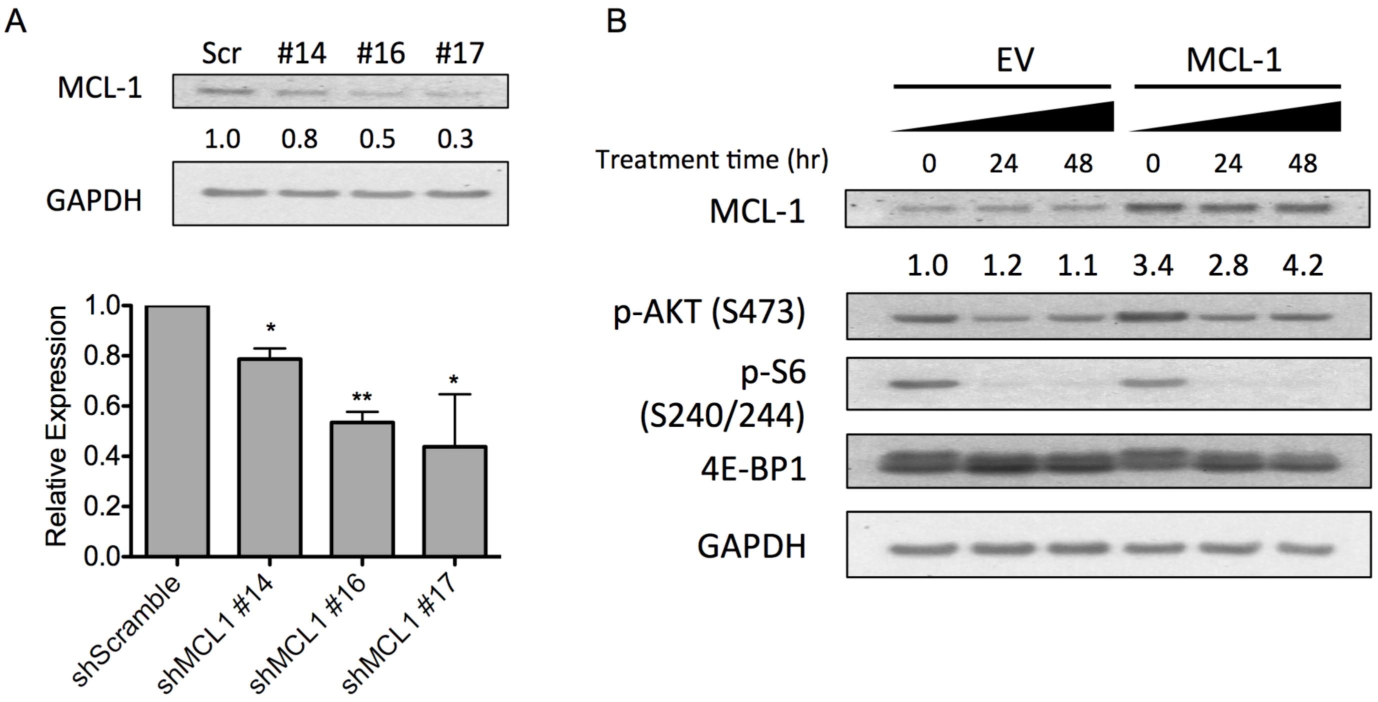

Supplemental Figure 6. Confirmation of MCL-1 knockdown and overexpression. (A) Representative immunoblot of MCL-1 knockdown in OCI-LY1 cells using three distinct shRNAs (upper) and quantification (lower). Densitometry values were normalized to GAPDH loading control, this ratio was then normalized to untreated empty vector cells. Knockdown was performed three separate times. Significance was determined using a two-tailed one-sample t test relative to normalized control. *$P < 0.05$, **$P < 0.005$. (B) Immunoblot analysis of MCL-1 expression in OCI-LY1 cells transduced with empty vector or a doxycycline-inducible MCL-1 expression vector. Cells were pre-treated with doxycycline (1 μg/ml) for 24 hours before treatment with BEZ235 for 24 or 48 hours. Data are representative of three independent experiments.

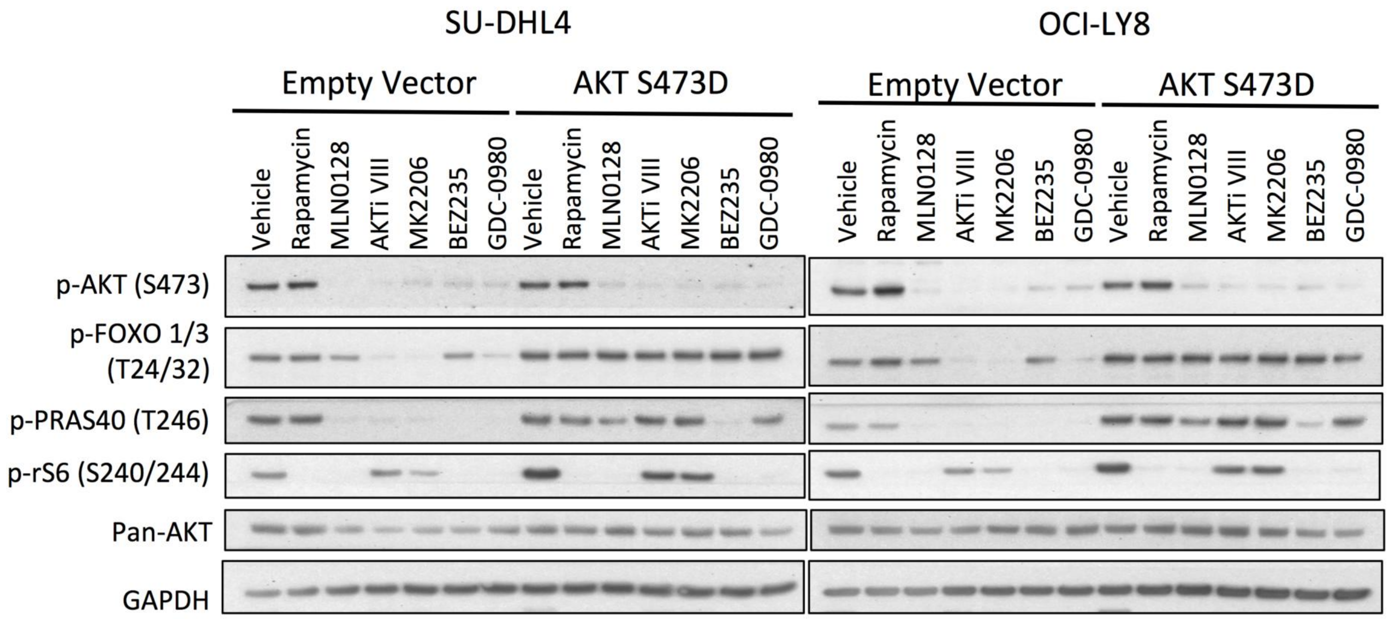

Supplemental Figure 7. Confirmation of expression of AKT S473D in SU-DHL4 and OCI-LY8 Cells. Immunoblot of SU-DHL4 and OCI-LY8 cells expressing either empty vector or phospho-mimetic AKT (S473D). Cells were pre-treated with doxycycline (1 µg/ml) for 24 hours prior to treatment with indicated PI3K pathway inhibitors for an additional 3 hours. Data are representative of three independent experiments.

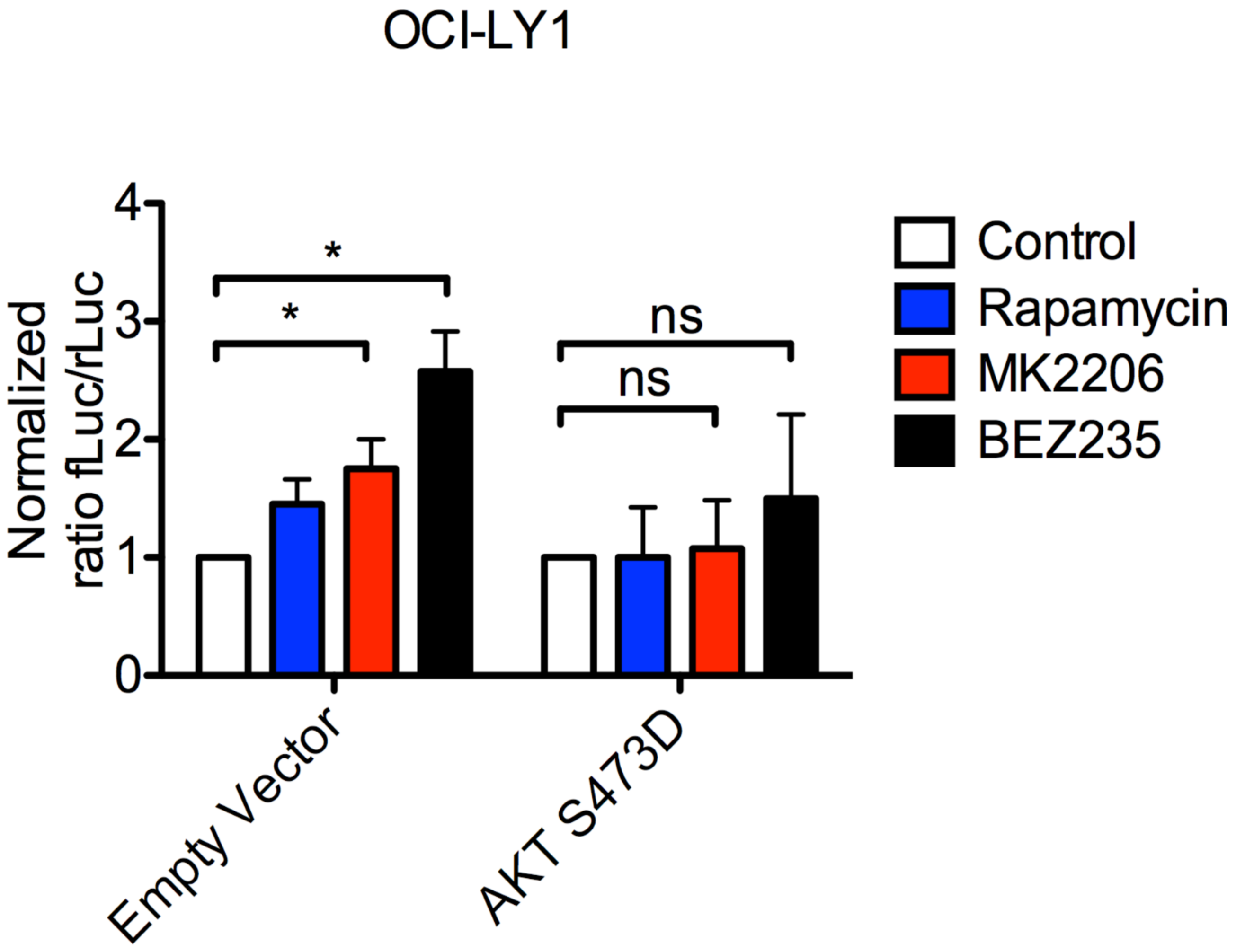

Supplemental Figure 8. Expression of AKT S473D suppresses FOXO activation following treatment with inhibitors of AKT or PI3K/mTOR. Relative FOXO transcriptional activity was measured using a luciferase reporter assay system. Cells were co-transfected with pRL-TK (renilla) and firefly luciferase downstream of the putative FOXO3 binding site prior to treatment with indicated inhibitor for 16 hours. The ratio of firefly (FOXO) to renilla (transfection control) luciferase activity was determined by luminometer, this value was then normalized to untreated control (n = 4). Data are shown as mean ± SD. Significance was calculated using one-tailed student's t test *$P < 0.05$.

Supplemental References

1. Schmidt EK, Clavarino G, Ceppi M, Pierre P. SUnSET, a nonradioactive method to monitor protein synthesis. Nature Methods. 2009; 6(4):275–7.

2. Jang GM, Leong LEC, Hoang LT, Wang PH, Gutman GA, Semler BL. Structurally distinct elements mediate internal ribosome entry within the 5'-noncoding region of a voltage-gated potassium channel mRNA. Journal of Biological Chemistry. 2004; 279(46):47419–30.
